# Computational approaches to treatment response prediction in major depression using brain activity and behavioral data: A systematic review

**DOI:** 10.1162/netn_a_00233

**Published:** 2022-10-01

**Authors:** Povilas Karvelis, Colleen E. Charlton, Shona G. Allohverdi, Peter Bedford, Daniel J. Hauke, Andreea O. Diaconescu

**Affiliations:** Krembil Centre for Neuroinformatics, Centre for Addiction and Mental Health (CAMH), Toronto, ON, Canada; Department of Psychiatry (UPK), University of Basel, Basel, Switzerland; Department of Mathematics and Computer Science, University of Basel, Basel, Switzerland; University of Toronto, Department of Psychiatry, Toronto, ON, Canada; Institute of Medical Sciences, University of Toronto, Toronto, ON, Canada; Department of Psychology, University of Toronto, Toronto, ON, Canada

**Keywords:** Major depressive disorder, Treatment response prediction, Machine learning, Computational psychiatry, fMRI, EEG

## Abstract

Major depressive disorder is a heterogeneous diagnostic category with multiple available treatments. With the goal of optimizing treatment selection, researchers are developing computational models that attempt to predict treatment response based on various pretreatment measures. In this paper, we review studies that use brain activity data to predict treatment response. Our aim is to highlight and clarify important methodological differences between various studies that relate to the incorporation of domain knowledge, specifically within two approaches delineated as data-driven and theory-driven. We argue that theory-driven generative modeling, which explicitly models information processing in the brain and thus can capture disease mechanisms, is a promising emerging approach that is only beginning to be utilized in treatment response prediction. The predictors extracted via such models could improve interpretability, which is critical for clinical decision-making. We also identify several methodological limitations across the reviewed studies and provide suggestions for addressing them. Namely, we consider problems with dichotomizing treatment outcomes, the importance of investigating more than one treatment in a given study for differential treatment response predictions, the need for a patient-centered approach for defining treatment outcomes, and finally, the use of internal and external validation methods for improving model generalizability.

## Author Summary

Individuals with major depressive disorder (MDD) vary in their response to available treatments, rendering treatment selection a challenging task. In this paper, we review studies applying computational models for predicting treatment response in MDD based on measures of brain activity. We discuss methodological differences across studies, focusing on how they incorporate existing knowledge about MDD and how that affects interpretability of model predictions. In this context, we argue that theory-driven generative modeling, which explicitly models information processing in the brain and thus can capture disease mechanisms, is a promising emerging approach for treatment response prediction. Finally, we identify several other important limitations that are holding back the translation of these tools into clinical practice.

## INTRODUCTION

Depressive disorders are the third highest cause of years lived with disability, affecting more than 264 million people worldwide (James et al., 2018). Major depressive disorder (MDD) is the most frequent type of depressive disorder (Vandeleur et al., 2017) and is characterized by depressed mood, diminished interests or pleasure, vegetative symptoms (e.g., appetite or sleep disturbances), and impaired cognition (e.g., feelings of worthlessness or inappropriate guilt; American Psychiatric Association, 2013). A key challenge in the treatment of MDD is the heterogeneity of illness course and treatment response (Luedtke & Kessler, 2021). Patients often show diverse initial symptoms with divergent disease trajectories over time, and some symptoms persist in spite of treatment (Rush et al., 2006). As a result, many patients face a long and painful trial-and-error process to identify the right treatment.

A promising way forward is to leverage computational models for understanding the heterogeneity of MDD and identifying individual predictors of differential treatment response. Broadly defined, computational psychiatry aims to formalize the relationship between the brain’s neurobiology, its environment, and psychiatric symptoms in computational terms (Adams, Huys, & Roiser, 2016). Within computational psychiatry, there are two conceptually different approaches: data-driven and theory-driven modeling (Huys, Maia, & Frank, 2016; Stephan et al., 2017). **Data-driven approaches** are domain-knowledge-agnostic, and classical statistics or machine learning techniques are used for exploratory analyses to discover predictive patterns in high-dimensional data. Conversely, **theory-driven approaches** use models that rely on domain-knowledge-derived hypotheses about the processes underlying neural and/or behavioral data. While advances in each approach have helped progress clinical research, each method comes with different trade-offs. Moreover, these approaches are not mutually exclusive and are often integrated in different ways. With this in mind, we review electroencephalography (EEG) and functional magnetic resonance imaging (fMRI) studies on MDD treatment response prediction and organize methodological differences among studies along the data-driven versus theory-driven dimensions. We limit our review to studies that predict treatment response using out-of-sample testing, while studies that report simple (in-sample) associations between the variables and treatment response outcomes will not be discussed.

In what follows, we first provide a brief introduction into data-driven and theory-driven approaches. We propose that the data-driven versus theory-driven trade-off could be seen along two dimensions related to the way data are collected and the way data are processed. This provides background for the following section, where we briefly summarize fMRI and EEG studies using data- or theory-driven methods for treatment response prediction in MDD, going from the least theory-driven to the most theory-driven approaches. Next, we present several promising theory-driven developments that are only beginning to be utilized for treatment response prediction in MDD. Finally, we discuss more generally what challenges are remaining for these tools to be translated into clinical practice.

## DIFFERENT TYPES OF APPROACHES

### Data-Driven Approaches

**Figure 1.**
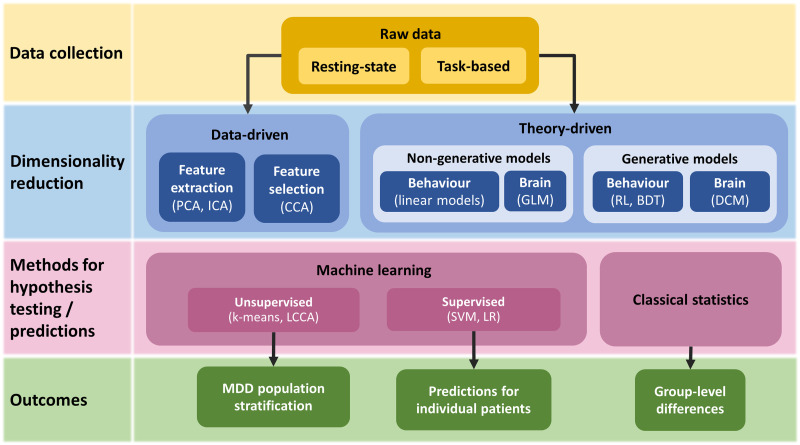
A conceptual overview illustrating common methods employed by data-driven and theory-driven approaches to study treatment response prediction in MDD. At the data collection stage, theory-driven knowledge can be incorporated via task-based designs, which probe specific cognitive functions. Most often, resting-state data will be processed with data-driven methods by performing feature selection, for example, using canonical correlation analysis (CCA), or feature extraction, for example, using principal or independent component analysis (PCA or ICA). Theory-driven dimensionality reduction is most often applied to task-based data using non-generative models, such as linear models used for obtaining summary statistics from behavioral data or the commonly used general linear model (GLM) in neuroimaging data analysis. A more advanced approach is to use generative models of behavior, such as reinforcement learning (RL) or Bayesian decision theory (BDT) models, which can be fit to behavioral data, or dynamic causal modeling (DCM), which can be applied to neuroimaging data. Next, the obtained features are used to train machine learning algorithms. Using supervised learning, such as support vector machines (SVM) or logistic regression (LR), one can determine the predictive ability of these features at the level of an individual patient. Unsupervised learning, such as k-means or latent class cluster analysis (LCCA), on the other hand, is primarily focused on stratifying MDD patient population, but the determined subpopulations could subsequently inform treatment response prediction. In contrast to machine learning approaches, classical statistics methods that are concerned with uncovering group-level effects (e.g., group differences between responders and nonresponders) and that do not provide predictions at the level of an individual patient are thus not included in this review.

Data-driven approaches aim to identify patterns or predictive relationships in high-dimensional datasets without relying directly on domain knowledge (Figure 1)—such as predicting treatment response based on neuroimaging data without requiring specific hypotheses about the neural mechanisms underlying MDD. To obtain discriminative features from the raw data, such approaches typically use either feature extraction or feature selection methods. Feature extraction involves constructing a smaller set of new features from the existing ones by applying techniques such as independent component analysis (ICA) or principal component analysis (PCA). Feature selection, on the other hand, does not transform the original features, but simply removes those that are irrelevant, such as highly intercorrelated features or features that do not correlate with the target variable (e.g., treatment response).

The resulting features are then used to train machine learning algorithms to predict treatment response. In this review, we consider two main types of machine learning algorithms: supervised and unsupervised. In **supervised learning**, an algorithm learns a function that maps input features (e.g., neuroimaging measures) to output labels (e.g., treatment outcome). Supervised algorithms are used for classification, the prediction of a discrete label (e.g., remission vs. nonremission), or regression, the prediction of a continuous variable (e.g., symptom improvement after treatment). Popular supervised classifiers include support vector machines (SVM; Cortes & Vapnik, 1995), logistic regression (Wright, 1995), and decision trees (e.g., Breiman, 2001). Comparatively, **unsupervised learning** does not require output labels, but instead finds patterns in the distribution of the input features. Clustering, the process of grouping data together based on underlying similarities, may be used to identify MDD subtypes or treatment response profiles, although ascertaining the clinical usefulness of the discovered clusters may require additional analyses with the data labels. As we will see in section *Studies Examining Treatment Response Prediction in MDD*, most of the neuroimaging studies on treatment response prediction in MDD to date have employed supervised learning methods.

### Theory-Driven Approaches

Theory-driven approaches employ domain-knowledge-informed techniques that utilize hypotheses about the underlying mechanisms of MDD (Figure 1). The models used in this approach can broadly be divided into generative and non-generative models. Non-generative models would subsume linear models used for obtaining summary statistics from behavioral data or the commonly used general linear model (GLM) in neuroimaging data analysis. Generally, such methods are used for exploratory analyses or to test hypotheses in terms of a specific contrast-, condition-, or group-related effect. In contrast, **generative models** aim to explicitly describe a mechanism that underlies neural or behavioral data in computational terms and thus are able to generate such data. These methods can span multiple levels of analyses, from biophysically informed models that describe the dynamics at a single neuron (e.g., ion channel conductances, membrane potential, firing rate), to generative models of brain responses that investigate experimentally induced coupling among brain areas, to generative models of behavior that describe information processing underlying decision-making. This type of approach allows for inference on disease mechanisms and provides a detailed model of the disease (Huys et al., 2016; Stephan et al., 2017). Another notable advantage of generative models is that they can be used to investigate the behavior of the system under different conditions through simulations. In this manner, simulations can be employed to generate new hypotheses about disease mechanisms and achieve a better understanding of the neurobiology of treatment effects (Stephan et al., 2017).

Through various (mostly non-generative) theory-driven approaches, MDD has been associated with deficits in reward processing and emotion regulation (Han, Ham, & Kim, 2021; Phillips et al., 2015), implicating serotonergic and dopaminergic neurocircuits, respectively (Keren et al., 2018; Kupfer, Frank, & Phillips, 2012; Pizzagalli, 2014). Some of the main findings involve elevated amygdala activation in response to negative emotional stimuli, increased activity of the anterior cingulate/ventromedial prefrontal cortex (ACC/vmPFC), which are involved in automatic emotion regulation, and attenuated activity of the dorsolateral PFC (dlPFC), which is involved in voluntary emotion regulation (Han et al., 2021; Phillips et al., 2015). With regards to reward processing, decreased activity of the ventral striatum has emerged as the most prominent finding (Keren et al., 2018). Large-scale network studies have also revealed increased connectivity within the affective network, reduced connectivity within the frontal-striatal reward network, diminished connectivity within the central executive network, and hyperconnectivity of the default mode network (DMN; Kaiser, Andrews-Hanna, Wager, & Pizzagalli, 2015; Li et al., 2018). Importantly, changes in each of these networks have been related to different symptoms (e.g., anhedonia being more associated with the reward network, and rumination with the DMN) and thus might differentially respond to treatments (Chahal, Gotlib, & Guyer, 2020; Li et al., 2018). Despite the increasing understanding of the neural mechanisms underlying MDD, recent review articles on regional activation and functional connectivity measures suggest, however, a lack of reliable neuromarkers for treatment response prediction (Fonseka, MacQueen, & Kennedy, 2018; Taylor, Kurt, & Anand, 2021), which highlights the need for methodological advancements.

### Combining Data-Driven and Theory-Driven Approaches

Data-driven and theory-driven approaches can be combined in different ways. This can be done along two dimensions: data collection and dimensionality reduction (Figure 1). In the former case, theory-driven knowledge is incorporated into the decision about what type of data is collected, for example, by probing cognitive mechanisms known to be implicated in MDD with cognitive tasks. In the latter case, theory-driven knowledge is incorporated by selecting a subset of data features (e.g., brain regions) or deriving the features based on previous literature or a priori hypotheses.

One of the main benefits of incorporating more theory-driven elements into treatment response prediction is the **interpretability** of the discriminative features (Finn, 2021; Stephan et al., 2017). Increased interpretability can help both clinicians and researchers better understand how model predictions and the associated neural markers relate to the behavioral and cognitive symptoms experienced by patients. For clinicians, interpretability is essential for making informed judgements based on a model’s prediction (Kelly, Karthikesalingam, Suleyman, Corrado, & King, 2019; Rudin, 2019). For researchers, interpretability can help them understand why certain treatments do not work for some individuals—which can provide insights for the development of new treatments (Stephan et al., 2017).

At the stage of data collection, interpretability can be improved by using **task-based neuroimaging** paradigms. Compared with **resting-state neuroimaging** protocols (which are more common in data-driven approaches), task-based paradigms are usually more sensitive to brain–behavior and brain–mind relationships (Finn, 2021). While resting-state data collection could also be motivated by specific hypotheses about rumination and mind wandering in MDD, and thus have a theory-driven motivation, without sufficient experimental control (e.g., experience sampling or retrospection) to relate the recorded brain activity to experience, treatment response predictions derived from such data can be difficult to interpret.

Interpretability can be further improved at the dimensionality reduction stage by applying theory-driven techniques, which incorporate knowledge or hypotheses about mechanisms underlying MDD. This is in contrast to data-driven dimensionality reduction, which does not require such knowledge and, as a result, offers less interpretable features. State-of-the-art theory-driven dimensionality reduction involves the use of generative models that describe information processing dynamics in the brain and by doing so is able to capture disease mechanisms in greater detail (Figure 1). Applying machine learning classification or clustering methods using features derived from fitting such generative models to data has been referred to as **generative embedding** (Brodersen et al., 2011; Frässle et al., 2018). However, this technique is only beginning to be utilized for treatment response prediction in MDD and is yet to prove its potential (see the section *Generative Modeling and Generative Embedding*).

## STUDIES EXAMINING TREATMENT RESPONSE PREDICTION IN MDD

In this section, we briefly summarize recent studies on treatment response prediction in MDD, going from the least to the most theory-driven approaches (Tables 1 and 2). Our aim is to highlight the variety of methodologies used and to draw attention to the important distinctions of data- versus theory-driven strategies (Figure 1). We restrict our review to studies that performed explicit analysis of treatment response prediction and incorporated validation techniques (e.g., **cross validation** or **external validation**) to increase the generalizability of findings. Studies that investigated associations between various neuromarkers and treatment response but did not use an out-of-sample validation to assess their predictive power are not discussed (for these studies, see recent reviews: Dichter, Gibbs, & Smoski, 2015; Fonseka et al., 2018; Kang & Cho, 2020; Olbrich & Arns, 2013; Phillips et al., 2015; Taylor et al., 2021). Additionally, in the interest of brevity, studies with sample sizes of 30 and below will not be discussed because of their limited generalizability (see Tables 1 and 2 for study summaries).

**Table 1.** Overview of data-driven MDD treatment response prediction studies. Relevant model performance metrics (BAC and *R*^2^) that were not reported by the studies but were possible to calculate from the reported values are included in parentheses. MDD - major depressive disorder, TRD - treatment-resistant depression, NDRI - norepinephrine-dopamine reuptake inhibitor, SSRI - selective serotonin reuptake inhibitor, SNRI - serotonin-norepinephrine reuptake inhibitor, rTMS - repetitive transcranial magnetic stimulation, tDCS - transcranial direct-current stimulation, ECT - electroconvulsive therapy, MADRS - Montgomery-Asberg Depression Rating Scale, HAMD - Hamilton Rating Scale for Depression, BDI - Beck Depression Inventory, QIDS-SR - Quick Inventory of Depressive Symptomatology—Self Report, ROI - region of interest, FC - functional connectivity, ICA - independent component analysis, SVM - support vector machine, LDA - linear discriminant analysis, CV - cross-validation, LOOCV - leave- one-out cross-validation, LOSOCV - leave-one-site-out cross-validation, Acc - accuracy, SE - sensitivity, SP - specificity, BAC - balanced accuracy, RMSE - root mean square error, dmPCF - dorsomedial prefrontal cortex, ACC - anterior cingulate cortex.

Reference	Subjects	Treatment	Modality	Outcome defintion	Features	Model	Validation	Performance
Jaworska et al. (2019)	51 MDD	NDRI (bupropion), SSRI (escitalopram), or combination of both	rsEEG	Response: ≥50% ↓ in MADRS	Demographics, baseline, & Week 1 clinical data, EEG power features, current source density	Random forest	10-fold CV	Acc 88%
								SE 77%
								SP 99%
Zhdanov et al. (2020)	122 MDD	SSRI (escitalopram)	rsEEG	Response: ≥50% ↓ in MADRS	Electrode-level & source-level spectral features, multiscale entropy-based & microstate-based features	SVM	Leave-one-site-out CV (LOSOCV)	BAC 79%
								SE 67%
								SP 91%
Khodayari-Rostamabad et al. (2010)	22 MDD	SSRI (mainly sertraline)	rsEEG	Response: ≥25% ↓ in HAMD-17	Spectral coherence, mutual information between electrode pairs, absolute & relative power spectral density	Kernel partial least squares regression	Nested CV	Acc 87%
								SE 88%
								SP 86%
Khodayari-Rostamabad et al. (2013)	22 TRD	SSRI (sertraline, citalopram, fluvoxamine, or paroxetine)	rsEEG	Response: ≥30% ↓ in HAMD-17	Power spectral density, squared spectral coherence, mutual information, left-to-right hemispheres, & anterior/posterior power ratio	Mixture of factor analysis	k-fold CV	Acc 88%
								SE 95%
								SP 81%
Rabinoff et al. (2011)	25 MDD	SSRI (fluoxetine) or SNRI (venlafaxine)	rsEEG	Response: HAMD-17 ≤ 10	Absolute & relative power, cordance features	Classification and regression trees (CART)	LOOCV	BAC 93%
								SE 85%
								SP 100%
Shahabi, Shalbaf, and Maghsoudi (2021)	30 MDD	SSRI (type not specified)	rsEEG	Response: ≥50% ↓ in BDI	3D images constructed from EEG signal	Convolutional neural networks	10-fold CV	Acc 97%
								SE 96%
								SP 97%
W. Wu et al. (2020)	109 MDD (sertraline), 119 MDD (placebo)	SSRI (sertraline)	rsEEG	Δ in HAMD-17	Theta, alpha, beta, gamma band power of latent signal	Linear regression	10-fold CV	(*R*^2^ = 0.36)
								*r* = 0.60
								RMSE = 5.68
								*p* = 2.88 × 10^−11^
Rajpurkar et al. (2020)	518 MDD	SSRI (escitalopram, sertraline) or SNRI (venlafaxine)	rsEEG	Δ in HAMD-21 (individual symptoms)	Absolute & relative power of delta, theta, alpha, beta, & gamma frequency bands in frontal & occipital regions	Gradient-boosted decision trees (GBDT)	5-fold stratified CV	Concordance index of ≥0.8 on 12 out of 21 symptoms
								*R*^2^ 0.3–0.7
Khodayari-Rostamabad, Reilly, Hasey, de Bruin, and MacCrimmon (2011)	27 TRD	rTMS	rsEEG	Response: ≥50% ↓ in HAMD-17	Anterior/posterior power ratios at various frequencies	Mixture of factor analysis	k-fold CV	Acc 80%
								(BAC 81%)
								SE 78%
								SP 83%
N. Bailey et al. (2019)	50 TRD	rTMS	rsEEG	Response: ≥50% ↓ in HAMD-17	Mood features, theta & alpha power & connectivity, frontal theta cordance & alpha peak frequency	SVM	5-fold CV	BAC 86%
								SE 84%
								SP 89%
Hasanzadeh et al. (2019)	46 MDD	rTMS	rsEEG	Response: ≥50% ↓ in HAMD-17 or BDI-II	Nonlinear, power spectral density, bispectrum, frontal & prefrontal cordance	k-nearest neighbors	LOOCV	BAC 91%
								SE 87%
								SP 96%
Al-Kaysi et al. (2017)	10 MDD	tDCS	rsEEG	Response: ≥50% ↓ in MADRS	Power spectral density in delta, theta, alpha, beta, & gamma frequency bands	SVM, LDA, extreme learning machine	LOOCV	Mood Labels Channels FC4-AF8: Acc 76%; Cognition Labels Channels CPz-CP2: Acc 92%
Tian et al. (2020)	106 MDD	SSRI (escitalopram)	rsfMRI	Response: ≥50% ↓ in HAMD-17	Multilayer modularity framework applied to the whole brain to obtain measures of functional integration & segregation among 95 ROIs	SVM	Leave-one-site-out CV (LOSOCV)	BAC 71%
Klöbl et al. (2020)	29 MDD	SSRI (escitalopram)	rsfMRI	Δ in HAMD-17; Response: ≥50% ↓ in HAMD-17; Remission: HAMD-17 ≤ 7	Whole-brain FC	Linear regression	k-fold CV	HAMD-sum: *r* = 0.51
								Response: BAC 60%, AUC 68%
								Remission: BAC 68%, AUC 73%
Chin Fatt et al. (2020)	132 MDD (sertraline), 132 MDD (placebo)	SSRI (sertraline)	rsfMRI	Δ in HAMD-17	Cortical & subcortical seed-based FC	Linear mixed model	LOOCV	(*R*^2^ = 0.05–0.13)
								*r* = 0.22–0.36
Korgaonkar et al. (2020)	163 MDD	SSRI (escitalopram, sertraline) or SNRI (venlafaxine)	rsfMRI	Remission: HAMD-17 ≤ 7	Whole-brain network intrinsic FC	Logistic regression	Hold-out test set	Average connectivity measures:
								Acc 69%
								(BAC 67%)
								SE 58%
								SP 76%
								Individual network connectivity:
								Acc 69%
								(BAC 68%)
								SE 63%
								SP 72%
Nemati et al. (2020)	99 MDD (sertraline), 103 MDD (placebo), & 19 MDD (ketamine), 19 MDD (active control), 18 MDD (inactive control)	SSRI (sertraline), ketamine	rsfMRI	Δ in HAMD-17	Network restricted connectivity	Network restricted strength predictive (linear) model	10-fold CV	Sertraline (vs. placebo): *r* = 0.27 (*R*^2^ = 0.07), *p* = 0.003; Ketamine (vs. active placebo): *r* = 0.57 (*R*^2^ = 0.32), *p* = 0.0002
Fan et al. (2020)	97 MDD (sertraline), 103 MDD (placebo)	SSRI (sertraline)	rsfMRI	Δ in HAMD-17 (%)	Network restricted connectivity	Network restricted strength predictive (linear) model	10-fold CV	Response to sertaline or placebo: (*R*^2^ = 0.04) *r* = 0.19, *p* = 0.03
Ju et al. (2020)	108 MDD	Various drugs; primarily: paroxetine, other SSRIs, sedative hypnotics, NDRI (bupropion)	rsEEG	Δ in HAMD-24	Whole-brain FC matrices	Connectome-based predictive modeling	LOOCV	*r* = 0.43 (*R*^2^ = 0.19), *p* = 2.73 × 10^−6^
Kong et al. (2021)	82 MDD	Antidepressants (type not specified)	rsfMRI	Response: >50% ↓ in HAMD-21	Dynamic functional networks	Spatiotemporal graph convolutional network	10-fold CV	Acc 90%
								(BAC 89%)
								SE 85%
								SP 93%
Drysdale et al. (2017)	154 MDD	rTMS	rsfMRI	Response: ≥50% ↓ in HAMD-17	Whole-brain FC matrices & biotype diagnosis	SVM	LOOCV	Only FC feature: Acc 78.3%
								FC features & biotype diagnosis: Acc 89.6%
Van Waarde et al. (2015)	45 severe/TRD	ECT	rsfMRI	Remission: MADRS score ≤ 10	Standard group ICA extracted 25 rs-networks. Each network was used to train a classifier	SVM	LOOCV	Two rs-networks had significant accuracy: **dmPFC**: BAC 85%; SE 84%; SP 85%; **ACC**: BAC 78%; SE 80%; SP 75%
Leaver et al. (2018)	46 TRD	ECT	rsfMRI, sMRI, arterial spin labeled fMRI	Response: average % improvement in HAMD-17, MADRS, and QIDS-SR. Split point was 42.2% reduction.	Mean voxelwise cerebral blood flow, regional homogeneity, fractional amplitude of low-frequency fluctuations, gray matter volume	SVM	Nested CV	BAC 58–68%
								SE 54–64%
								SP 55–74%
Sun et al. (2020)	122 MDD or bipolar disorder	ECT	rsfMRI	Δ in HAMD-17; Remission: HAMD-17 score < 7	Negatively & positively correlated FC networks based on whole-brain rsFC	Linear regression	LOOCV	Negative FC networks:
								*r* = 0.51
								(*R*^2^ = 0.26)
								Acc 76%
								(BAC 72%)
								SE 51%
								SP 92%

**Table 2.** Overview of data- and theory-driven MDD treatment response prediction studies. Relevant model performance metrics (BAC and *R*^2^) that were not reported by the studies but were possible to calculate from the reported values are included in parentheses. MDD - major depressive disorder, TRD - treatment-resistant depression, LLD - late-life depression, SSRI - selective serotonin reuptake inhibitor, rTMS - repetitive transcranial magnetic stimulation, SNRI - serotonin-norepinephrine reuptake inhibitor, NDRI - norepinephrine-dopamine reuptake inhibitor, CBT - cognitive behavioral therapy, ECT - electroconvulsive therapy, BDI - Beck Depression Inventory, HAMD - Hamilton Rating Scale for Depression, MADRS - Montgomery-Asberg Depression Rating Scale, QIDS-SR - Quick Inventory of Depressive Symptomatology—Self Report, SOFAS - Social and Occupational Functioning Assessment Scale, CIDI - Composite International Diagnostic Interview, LCI - Life Chart Interview, STFT - short-time Fourier transform, EMD - empirical mode decompositions, ACC - anterior cingulate cortex, FC - functional connectivity, ROI - region of interest, ICA - independent component analysis, BOLD - blood oxygen level dependent, dlPFC - dorsolateral prefrontal cortex, amPFC - anterior medial prefrontal cortex, mPFC - medial prefrontal cortex, DMN - default mode network, SN - salience network, PCC - posterior cingulate cortex, AN - affective network, VIS - visual, SVM - support vector machine, ROC - receiver operating characteristic, CV - cross-validation, LOOCV - leave-one-out cross-validation, Acc - accuracy, SE - sensitivity, SP - specificity, BAC - balanced accuracy, AUC - area under curve, RMSE - root mean square error.

Reference	Subjects	Treatment	Modality	Outcome definition	Features	Model	Validation	Performance
Mumtaz et al. (2017)	34 MDD	SSRIs (type not specified)	tbEEG: 3-stimulus visual oddball task	Response: ≥50% ↓ in BDI-II	Combination of wavelet coefficients, STFT, & EMD features	Logistic regression	10-fold CV	Acc 92%
								SE 90%
								SP 90%
N. Bailey et al. (2018)	39 TRD	rTMS	tbEEG; Working memory task	Response: ≥50% ↓ in HAMD-17	Baseline and Week 1 MADRS scores, task accuracy & reaction time, alpha, theta, gamma power & connectivity, theta gamma coupling	SVM	5-fold CV	BAC 91%
								SE 90%
								SP 92%
Miller et al. (2013)	17 MDD	SSRI (escitalopram)	tbfMRI; emotional words task	Δ in HAMD-24	Clusters whose active during negative word processing was associated with treatment outcome	Linear regression	10-fold CV	(*R*^2^ = 0.23) *r* = 0.48, *p* < 0.05
Godlewska et al. (2018)	32 MDD	SSRI (escitalopram)	tbfMRI: emotional faces task	Response: ≥50% ↓ in HAMD-17	Mean cluster activity within ACC for sad vs. happy faces	Single-feature ROC	LOOCV	BAC 72%
Fu et al. (2008)	19 MDD	SSRI (fluoxetine)	tbfMRI; implicit sad facial affect recognition task	Remission: HAMD-17 ≤ 8	Whole-brain FC for each facial expression intensity (low, medium, high)	SVM	LOOCV	Low intensity of sad facial expressions:
								SE 75%
								SP 62%
								*p* value = 0.11 (n.s.)
Fonzo et al. (2019)	115 MDD (sertraline), 122 MDD (placebo)	SSRI (sertraline)	tbfMRI; Emotional conflict task	Δ in HAMD-17	incongruent trials–congruent trials brain activation in several ROIs	Relevance vector machine	10 × 10 stratified CV	(*R*^2^ = 0.24) *r* = 0.49, *p* < 0.001
Karim et al. (2018)	49 LLD	SNRI (venlafaxine)	rsfMRI and tbfMRI; emotional regulation & emotional reactivity task	Remission: MADRS ≤ 10	Active regions during emotional reactivity, emotion regulation, or centrality at baseline & after single dose	Logistic regression	10-fold CV	(BAC 70%)
								AUC 77%
								SE 72%
								SP 68%
Williams et al. (2015)	80 MDD	SSRI (escitalopram, sertraline) or SNRI (venlafaxine)	tbfMRI; Supraliminal & subliminal facial emotion task	Response: ≥50% ↓ in HAMD-17	Emotion vs. neutral amygdala activation	Discriminant analysis	LOOCV	All medication types: Acc 75%
								SNRI only: Acc 77%
Goldstein-Piekarski et al. (2016)	70 MDD	SSRI (escitalopram, sertraline) or SNRI (venlafaxine)	tbfMRI; emotional faces task	Functional remission: HAMD-17 ≤ 7 and QIDS-SR ≤5 and ≥10 improvement to achieve ≥61 on SOFAS	Early life stress & anygdala reactivity	Logistic regression	LOOCV	(BAC 77%)
								SE 84%
								SP 69%
								AUC 81%
Crane et al. (2017)	29 MDD	SSRI (escitalopram) or SNRI (duloxetine)	tbfMRI; Go/No-Go task	Remission: HAMD score < 8	No-Go accuracy, two ICA component beta weights, & within-component clusters	Logistic regression, Random forest	LOOCV	Logistic regression:
								Acc 90%
								(BAC 90%)
								SE 90%
								SP 89%
								Random forest:
								Acc 84%
								BAC (82%)
								SE 84%
								SP 80%
Tozzi et al. (2020)	124 MDD	SSRI (escitalopram, sertraline) or SNRI (venlafaxine)	tbfMRI; Go/No-Go task	Response: >50% ↓ in QIDS-SR-16	ROIs with BOLD response in No-Go > Go condition in MDD patients & healthy controls	Logistic regression	LOOCV	Venlafaxine response:
								BAC 79%
								SE 67%
								SP 89%;
								Sertraline response:
								BAC 84%
								SE 95%
								SP 74%
Marquand, Mourão-Miranda, Brammer, Cleare and Fu (2008)	20 MDD	SSRI (fluoxetine)	tbfMRI; n-back task	Response: ≥50% ↓ in HAMD-17	Principal components based on whole-brain activity for each task condition	SVM	LOOCV	BAC 69%
								SE 85%
								SP 52%
Meyer et al. (2019)	22 MDD	SSRI (escitalopram)	tbfMRI; n-back task	Remission: MADRS ≤ 5 Nonremission: MADRS ≥ 10	dlPFC, amPFC, & parietal lobe	Single-feature ROC	LOOCV	(BAC 87%)
								AUC 85%
								SE 82%
								SP 91%
Nguyen et al. (2019)	37 MDD	NDRI (bupropion)	tbfMRI; reward processing task	Δ in HAMD-17; Remission: HAMD-17 score < 7	ROIs from anticipation contrast maps & reward expectation contrast maps	Dense feedforward neural networks	3 × 3 nested CV	*R*^2^ = 0.26
								RMSE = 4.71
								AUC 71%
Brandt et al. (2021)	90 MDD	SSRI (escitalopram); an optional switch to SNRI (duloxetine) from Week 4	tbfMRI; reward processing task	HAMD-6 Remission: >50% ↓ at week 4 *and* <5 at Week 8; Nonresponse: <25% ↓ a Week 4 *and* <50% ↓ at Week 8	Age, sex, baseline HAMD-6 and number of omissions in the task, & reward-related brain responses in striatum, anterior insula, & mPFC	Logistic regression	5-fold CV	AUC 56%
Costafreda, Khanna, Mourao-Miranda, and Fu (2009)	16 MDD	CBT	tbfMRI; sad facial expression task	Remission: HAMD-17 ≤ 7	Principal components based on whole-brain activity to viewing sad faces	SVM	LOOCV	BAC 79%
								SE 71%
								SP 86%
Cook et al. (2020)	129 MDD	SSRI (escitalopram) or NDRI (bupropion)	rsEEG	Remission: HAMD-17 ≤ 7	Theta and alpha power values from FT7-FPz and FT8-FPz channels at baseline and Week 1 (QEEG)	Single-feature ROC analysis	Jack-knife CV	Escitalopram remission:
								Acc 64%
								(BAC 65%)
								SE 74%
								SP 55%
Erguzel et al. (2015)	55 TRD	rTMS	rsEEG	Response: ≥50% ↓ in HAMD-17	Frontal cordance calculated from delta & theta bands (QEEG)	Artificial neural network	k-fold CV	Acc 85–89%
								(BAC 86–89%)
								SE 87–94%
								SP 84%
								AUC 87–91%
Patel et al. (2015)	33 LLD	SSRI (escilatopram) or SNRIs (duloxetine or venlafaxine)	rsfMRI; sMRI	Response: HAMD score < 10	Demographics, cognitive ability scores, functional connectivity index of dorsal DMN and anterior SN, & structural imaging variables	Alternating decision tree (ADTree)	Nested LOOCV	Acc 89%
								SE 89%
								SP 90%
Pei et al. (2020)	98 MDD	SSRIs (mainly escitalopram) & SNRIs (mainly venlafaxine)	rsfMRI	Response: ≥50% ↓ in HAMD-6	14 priori brain regions of interest based on previous literature	SVM	LOOCV	BAC 81%
								SE 78%
								SP 84%
Sikora et al. (2016)	29 MDD	1-week placebo & 10-week open-label antidepressant (SSRI, SNRI, NDRI, atypical)	rsfMRI	Δ in QIDS	Baseline SN rsFC	Multivariate relevance vector regression	LOOCV	Placebo response: (*R*^2^ = 0.17) *R* = 0.41, *p* value = 0.018; Antidepressant response: *r* = 0.03, *p* value = 0.340
Braund et al. (2022)	229 MDD	SSRI (escitalopram, sertraline) or SNRI (venlafaxine)	rsfMRI	Response: ≥50% ↓ in HAMD-17 or QIDS-SR	Whole-brain network intrinsic FC associated with neuroticism	SVM	LOOCV	Acc 75%
								(BAC 74%)
								SE 63%
								SP 85%
								AUC 76%
Goldstein-Piekarski et al. (2018)	75 MDD	SSRI (escitalopram, sertraline) or SNRI (venlafaxine)	rsfMRI	Remission: HAMD-17 ≤ 7	Seed-based posterior cingulate cortex	Logistic regression	LOOCV	PCC–ACC/mPFC:
								(BAC 78%)
								SE 73%
								SP 82%
								AUC 77%
C. T. Wu et al. (2018)	22 TRD	Sham-rTMS	rsfMRI	Δ in HAMD-17	Two feature sets: 1. Global brain activity; 2. Rostral ACC seed-based	Elastic-net regression	LOOCV	Global brain activity: (*R*^2^ = 0.24) *r* = 0.49; *p* = 0.023; rostral ACC FC: (*R*^2^ = 0.25) *r* = 0.50; *p* = 0.018
Cash et al. (2019)	47 MDD	rTMS	rsfMRI	Response: ≥25% ↓ in MADRS; Δ in MADRS	rsFC within the DMN & AN	SVM	LOOCV, k-fold CV	Acc 93%
								SE 95%
								SP 92 %
								(*R*^2^ = 0.46)
								*r* = 0.68
								*p* value < 0.001
Hopman et al. (2021)	70 TRD	rTMS	rsfMRI	Response: ≥50% ↓ in MADRS	Seed-based analysis with left dlPFC and subgenual ACC	SVM	Nested CV	Acc 89%
Moreno-Ortega et al. (2019)	18 TRD	ECT	rsfMRI	Remission: 24-HAMD score ≤ 7	dlPFC, DMN, & VIS networks	Logistic regression	LOOCV	Acc 89%
Frässle et al. (2020)	85 MDD	SSRIs or no treatment	tbfMRI; emotional face perception task	Long-term outcomes: remitted vs. improved vs. chronic, based on a latent class growth analysis using CIDI and LCI	Effective connectivity pattern in the network mediating emotional face perception	SVM	Nested LOOCV	Chronic vs. remitted:
								AUC 87%
								BAC 79%
								SE 97%
								SP 60%
								Improved vs. remitted:
								AUC 63%
								BAC 61%
								SE 77%
								SP 45%
Queirazza et al. (2019)	25 MDD	Computerized CBT	model-based fMRI; RL task	Response: >50% ↓ in BDI-II	Neural activity encoding weighted reward prediction errors	SVM	LOOCV	AUC 82%
								BAC 72%
								SE 63%
								SP 83%

**Figure 2.**
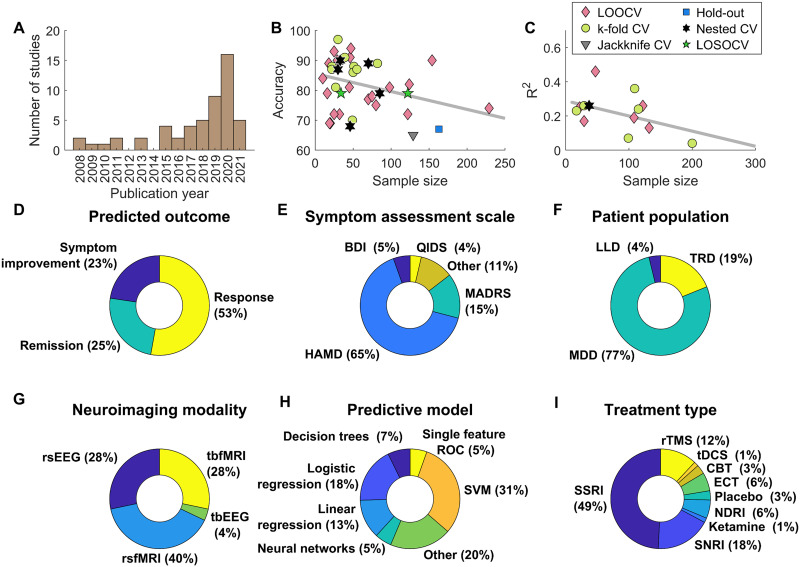
A visual summary of the reviewed studies. (A) The number of studies published each year. (B) Reported outcome prediction accuracy and (C) coefficient of determination as a function of sample size and broken down by validation methods used. LOOCV: leave-one-out cross-validation. LOSOCV: leave-one-site-out cross-validation. (C–I) Statistics of the reviewed 53 studies on the relevant metrics of study design. (D) The definition of outcomes. Symptom improvement: predicting symptom improvement after treatment on a continuous scale. Remission: predicting whether a certain threshold (e.g., ≤7 total score on the 17-item Hamilton Rating Scale for Depression, HAMD-17, after treatment) will be reached. Response: predicting whether a certain amount of reduction in symptoms will be reached (e.g., ≥50% reduction in the total HAMD score after treatment). (E) The scale used to assess depressive symptoms. (F) The studied patient population. MDD: major depressive disorder. TRD: treatment-resistant depression. LLD: late-life depression. (G) The neuroimaging modality used for data collection. rs: resting state. tb: task-based. (H) The model class used for treatment response prediction. ROC: receiver operating characteristic. (I) The treatment type for which predictions were made. SSRI: selective serotonin reuptake inhibitor. SNRI: serotonin-norepinephrine reuptake inhibitor. NDRI: norepinephrine-dopamine reuptake inhibitor. tDCS: transcranial direct-current stimulation. rTMS: repetitive transcranial magnetic stimulation. ECT: electroconvulsive therapy. CBT: cognitive behavioral therapy.

The literature search was performed during the period of June to October 2021. Using the search terms depress* AND treatment response prediction AND (accuracy OR sensitivity OR specificity OR regression) AND (fMRI OR EEG) on PubMed yielded 328 articles. Following a double rater assessment procedure, 35 articles met the inclusion criteria, with the main reasons for exclusion being (a) reviews and meta-analyses; (b) study protocols; (c) studies investigating other disorders; and (d) studies using no validation techniques. A further literature search was conducted using additional sources: (a) reference lists of already qualifying papers and related reviews, and (b) a search engine ResearchRabbit (www.researchrabbit.ai), which allows for the discovery of the most related papers based on a collection of input papers. This revealed 18 additional articles, leading to a total of 53 articles, which are reviewed below (Figure 2, Tables 1 and 2).

### Strongly Data-Driven Studies

#### rsEEG.

Resting-state EEG (rsEEG) studies are used to evaluate intrinsic neural activity, which is not elicited through a specific task. Resting-state measures often require less domain-specific knowledge, while EEG itself is inexpensive and quick to administer. EEG signals are often classified into frequency bands (i.e., delta, theta, alpha, beta, and gamma), each of which have been associated with different brain states (e.g., sleep, rest, alertness). Common EEG measures used as features for treatment response prediction include absolute and relative power, as well as coherence of frequency bands, and the majority of data-driven rsEEG studies have focused on predicting first-line antidepressant response (i.e., selective serotonin reuptake inhibitors, SSRIs).

While features from all frequency bands may be valuable for predicting first-line antidepressant response, the most predictive features tend to be from either alpha and/or theta bands. A recent study by Jaworska, de la Salle, Ibrahim, Blier, and Knott (2019) found alpha and theta power in the frontoparietal area to be highly predictive of SSRI (escitalopram) and norepinephrine–dopamine reuptake inhibitor (NDRI) (bupropion) response, with clinical, EEG, and current source density measures achieving 88% accuracy using an SVM classifier. Zhdanov et al. (2020) demonstrated similar results, whereby high alpha band power in the ACC was highly predictive of escitalopram response. An SVM classifier trained on clinical and EEG features with leave-one-site-out cross-validation (LOSOCV) yielded a slightly lower balanced accuracy (BAC) of 79%.

W. Wu et al. (2020) and Rajpurkar et al. (2020) analyzed rsEEG data from two large multisite clinical trials for depression: EMBARC (Establishing Moderators and Biosignatures of Antidepressant Response in Clinic Care), and iSPOT-D (International Study to Predict Optimized Treatment for Depression), respectively. W. Wu et al. (2020) introduced their Sparse EEG Latent SpacE Regression algorithm to predict sertraline response, and found that only alpha band power—and not theta, beta, or gamma—significantly predicted symptom improvement (*r* = 0.60, *p* = 2.88 × 10^*−*11^). The same model could not predict symptom improvement in the placebo group, suggesting that the model captured features unique to the antidepressant response. The authors further validated the model on an independent rsEEG dataset from Fonzo et al. (2019), which yielded a similar classification performance based on fMRI recordings (*r* = 0.44, *p* = 0.02; see the section *Theory-Driven Task Design* for discussion on the study), thus providing evidence for a neurobiological phenotype that can be detected across neuroimaging modalities. Rajpurkar et al. (2020) predicted *individual* symptom improvement in response to three SSRIs (escitalopram, sertraline, or venlafaxine). Baseline symptom scores and relevant EEG features were used to train a gradient-boosted decision tree model achieving a concordance index of ≥0.8 on 12 out of 21 clinician-rated symptoms (*R*^2^ 0.3–0.7). Concordance index indicates the probability that, given two random patients, the algorithm will correctly identify which patient showed greater improvement. Surprisingly, the inclusion of treatment groups did not significantly improve model performance, suggesting that the EEG markers were general predictors of treatment outcome instead of predictors of differential treatment response.

For patients who fail to respond to first-line antidepressants, or who are unable to tolerate medications, repetitive transcranial magnetic stimulation (rTMS) may be used as an alternative treatment. N. Bailey et al. (2019) used primarily alpha and theta frequency band features to predict rTMS response, achieving 87% accuracy, and found that rTMS responders showed elevated theta connectivity and lower alpha power at baseline and Week 1, while nonresponders showed typical theta connectivity (similar to that of controls). However, the same group later attempted to replicate these results using a large independent dataset (*N* = 193), and found no significant difference in theta connectivity or alpha power between rTMS responders and nonresponders (N. W. Bailey et al., 2021). Hasanzadeh, Mohebbi, and Rostami (2019) used a combination of power spectrum features across five frequency bands to predict rTMS response in MDD, achieving 91% BAC, and found that rTMS responders had significantly lower baseline beta power.

#### rsfMRI.

rsfMRI measures spontaneous brain activity believed to reflect functional communication between spatially distributed brain regions (van den Heuvel & Pol, 2010). Growing literature supports the notion that depression is associated with widespread aberrant functional connectivity, mainly in frontostriatal and limbic brain networks (Greicius et al., 2007; Mayberg et al., 2005; Sheline et al., 2009). Hence resting-state functional connectivity (rsFC) patterns, either in specific networks or on a whole-brain scale, can provide a platform for investigating the hypothesized functional disconnectivity effects in MDD and network changes in response to treatment.

Similar to rsEEG, a majority of rsfMRI studies investigated the impact of first-line pharmacotherapeutic interventions, and hyperconnectivity in the default mode network (DMN) was a common emergent finding across studies. A multisite study by Tian et al. (2020) applied a multilayer modularity framework with leave-one-site-out CV to predict SSRI (escitalopram) response using whole-brain features of functional segregation and integration, achieving accuracy rates of 69–72% across sites. Treatment responders showed stronger connections between the ACC and nodes within the DMN, suggesting that high interactions of the ACC with other regions may be predictive of treatment response. Chin Fatt et al. (2020) predicted SSRI (sertraline) versus placebo response using major cortical rs-networks and subcortial regions with a linear mixed model (*r* = 0.22–0.36). In general, the authors found that higher connectivity within the DMN and between the DMN and the executive control network predicted better treatment outcome. Using data from the international iSPOT-D trial, Korgaonkar, Goldstein-Piekarski, Fornito, and Williams (2020) predicted SSRI (escitalopram or sertraline) and SNRI (venlafaxine) response using intrinsic FC derived from task-based fMRI data. The authors found that irrespective of medication type, greater connectivity within the DMN was predictive of treatment remission (BAC 68%). In comparison, using a functional connectome “fingerprint” at one week posttreatment, Nemati et al. (2020) found that, compared with placebo, reductions in the DMN predicted better response to sertraline (*r* = 0.27), suggesting an early pattern of normalization in the DMN. Furthermore, the authors investigated the generalizability of the sertraline connectome fingerprint in predicting ketamine (rapid antidepressant) response. The model predicted ketamine response compared with an active control (lanicemine; *r* = 0.57), but failed to predict ketamine response compared with placebo (*p* > 0.05). Using the same study sample, Fan et al. (2020) identified a baseline functional connectome fingerprint that significantly predicted symptom improvement irrespective of treatment type (sertraline or placebo), but unlike Nemati et al. (2020), failed to predict the antidepressant treatment-specific response.

A couple of studies used whole-brain rsFC to investigate treatment response in mixed-treatment MDD cohorts (medications included SSRIs, SNRIs, and NDRIs). Ju et al. (2020) used a linear regression model and significantly predicted symptom improvement (*r* = 0.43, *p* = 2.73 × 10^−6^) at one month, and in out-of-sample patients for up to three months. Kong et al. (2021) developed a novel spatiotemporal graph convolutional network (STGCN) framework, which predicted treatment response with 90% accuracy.

A study by Drysdale et al. (2017) used whole-brain resting-state networks to investigate differential rTMS response between four depressive subtypes, which are neurophysiological subtypes defined by distinct patterns of dysfunctional connectivity in limbic and frontostriatal networks. Using connectivity features and this biotype classification, an SVM classifier was able to predict rTMS response with 90% accuracy. The most discriminating connectivity features involved the dorsomedial prefrontal (dmPFC) stimulation target and the left amygdala. The final model was further validated on an independent replication set (*n* = 30) and obtained comparable accuracy rates (88–93%). However, when Dinga et al. (2019) attempted to replicate depressive subtypes identified by Drysdale and colleagues on an independent sample (*n* = 187), the authors were unable to replicate these findings and found the methodology to be unreliable in their sample.

Finally, several studies have investigated rsFC patterns associated with electroconvulsive therapy (ECT) response. Van Waarde et al. (2015) used standard group ICA to extract rs-networks, each of which trained an SVM classifier, and found that rs-networks centered in the dmPFC (BAC = 85%) and ACC (BAC = 78%) significantly predicted ECT response in severe and treatment-resistant depression. Leaver et al. (2018) used multimodal fMRI metrics to predict ECT response achieving significant BACs (58–68%). Notably, the left dlPFC and subgenual ACC, both targets of rTMS, as well as connectivity between motor and temporal networks (near ECT electrodes), were consistently identified as informative features in the models. Finally, a larger study (*n* = 122) by Sun et al. (2020) used whole-brain rsFC to train a connectome-based model to predict depressive rating changes and remission status following ECT. Negative FC networks (anti-correlated with changes in depressive scores) were the most predictive (*r* = 0.51, accuracy = 76%), with FC between the inferior frontal gyrus and temporal regions demonstrating the most predictive power.

### Combination of Data-Driven and Theory-Driven Methods

Up until now we have discussed studies that relied solely on data-driven approaches throughout the analysis pipeline. In this section, we will discuss studies that combined data-driven and theory-driven methods by considering two dimensions: data collection and dimensionality reduction.

#### Theory-driven task design.

Several studies have incorporated domain knowledge at the level of the study design itself, that is, by easuring brain activity during cognitive tasks that probe specific mechanisms previously shown to be implicated in MDD. Broadly, these mechanisms relate to reward processing and emotion regulation (Han et al., 2021; Phillips et al., 2015; Stuhrmann, Suslow, & Dannlowski, 2011).

##### Task-Based EEG.

Using an EEG visual oddball task to predict SSRI response, Mumtaz, Xia, Mohd Yasin, Azhar Ali, and Malik (2017) compared three time-frequency decomposition techniques for feature extraction. EEG-based wavelet features extracted from frontal and temporal areas involving delta and theta frequency bands were the most predictive, with a logistic regression model producing an accuracy of 92%. N. Bailey et al. (2018) employed an EEG working memory task to evaluate rTMS response in patients with treatment-resistant depression achieving 91% BAC with an SVM classifier. At baseline and Week 1, responders showed enhanced fronto-midline theta power and higher theta connectivity compared with nonresponders. Although, using rsEEG data, increased theta connectivity in rTMS responders was later replicated by the same group (N. Bailey et al., 2019), this finding was subsequently disproved using a large independent dataset (N. W. Bailey et al., 2021).

##### Task-Based fMRI.

The majority of task-based studies employed emotional paradigms to probe abnormal processing of emotional stimuli commonly implicated in MDD (Stuhrmann et al., 2011). Using an emotional faces task, Godlewska et al. (2018) predicted SSRI (escitalopram) response based on the mean ACC activity to sad versus happy facial expressions. A moderate accuracy of 72% was achieved with responders showing increased pretreatment pregenual ACC activity to sad versus happy faces. Fonzo et al. (2019) used an emotional conflict task to predict symptom improvement following SSRI (sertraline) treatment and found that a greater downregulation of conflict-responsive regions predicted better outcomes (*r* = 0.49, *p* < 0.001). The same model could not predict improvement in the placebo group, suggesting that the model captured features unique to the antidepressant. Karim et al. (2018) employed an emotion regulation and emotion reactivity task, as well as rsfMRI to predict SNRI (venlafaxine) response in late-life depression (LLD). Using whole-brain connectivity and regions of task activation at baseline and one day following treatment, a logistic regression model achieved an AUC (area under the curve) of 77%, outperforming the use of baseline fMRI alone. The majority of predictive regions were in the frontal cortex, with the emotional reactivity task producing the most informative features.

Two studies assessed patients from the iSPOT-D trial (SSRI/SNRI treatment), whereby an emotional faces paradigm was used to investigate amygdala reactivity. Williams et al. (2015) found that amygdala hyporeactivity to subliminal happy and threat expressions was a general predictor of treatment response (accuracy 75%). However, amygdala reactivity to subliminal sadness functioned as a differential predictor, whereby nonresponders to SNRIs showed pretreatment hyperreactivity to subliminal sadness, which progressed to hyporeactivity posttreatment, and predicted SNRI response with 77% accuracy. Goldstein-Piekarski et al. (2016) investigated the interaction between amygdala engagement and early life stress to predict “functional remission,” which the authors defined by combining measures of the clinician-rated HAMD, self-reported 16-item Quick Inventory of Depressive Symptomatology–Self-Rated (QIDS-SR16), and observer-rated functional capacity using the Social and Occupational Functioning Assessment Scale. A discriminant analysis yielded a BAC of 77%, and similar to Williams et al. (2015), in patients with low early life stress, lower amygdala reactivity to both happy and threat-related stimuli increased the likelihood of remission. In comparison, for those with high exposure to early life stress, greater amygdala reactivity to happy stimuli predicted remission.

Using a Go/No-Go task, Tozzi, Goldstein-Piekarski, Korgaonkar, and Williams (2020) investigated differential response to SSRIs (escitalopram and sertraline) and SNRIs (venlafaxine). Connectivity between the dlPFC and the supramarginal gyrus (SMG) and between SMG and the middle temporal gyrus (MTG) was associated with response to sertraline and venlafaxine, but not to escitalopram. Using baseline symptom scores and the mean FC contrast values as inputs to a logistic regression classifier, venlafaxine response was predicted with BAC of 79%, while sertraline response was predicted with BAC of 84%. Interestingly, higher FC between both dlPFC-SMG and SMG-MTG was associated with response to sertraline, whereas lower connectivity was associated with response to venlafaxine.

Nguyen et al. (2019) employed a reward processing task and deep learning model to predict depressive rating changes and response status following NDRI (bupropion) treatment. Regions of activation were extracted from two contrast maps, one for anticipation and the other for reward expectation. The final model achieved a root mean square error of 4.71 (*R*^2^ = 0.26, AUC = 71%), and important clusters included the medial frontal cortex, amygdala, cingulate cortex, and striatum. The final model performed poorly (negative *R*^2^) on SSRI (sertraline) and placebo-treated subjects from the same dataset, suggesting that the model likely learned features specific to the bupropion response. Finally, a recent study by Brandt et al. (2021) used a comparatively large MDD cohort (*n* = 90) and found that pretreatment reward-related brain activity was not predictive of SSRI (escitalopram) treatment response. The authors also found no differences in reward reactivity estimates between healthy and depressed individuals and no change following eight weeks of treatment.

#### Theory-driven feature selection

##### rsEEG.

Two studies used quantitative EEG and theory-driven feature selection, particularly measures in the theta band over frontal regions, to predict treatment outcome. Cook, Hunter, Caudill, Abrams, and Leuchter (2020) predicted SSRI (escitalopram) and NDRI (bupropion) remission using a previously validated biomarker, the Antidepressant Treatment Response (ATR) index (Cook et al., 2020; Leuchter et al., 2009), which combines theta and alpha power metrics at baseline and Week 1 in frontotemporal channels. Higher ATR values were predictive of SSRI remission (BAC 65%), but not NDRI remission, which was selected as a control comparison. Erguzel et al. (2015) used an artificial neural network to predict rTMS response in patients concurrently receiving SSRIs, and using frontal cordance values from theta and delta bands, the model achieved BACs of 86–89%.

##### rsfMRI.

Based on previous literature, two studies selected a subset of resting-state features for the prediction of SSRI and SNRI response. In late-life depression, Patel et al. (2015) employed an Alternating Decision Tree model to predict antidepressant response yielding 89% accuracy based on cognitive scores, structural, and rsFC features (in the default mode network, DMN, and anterior salience network, aSN). Fewer structural connections in the aSN was predictive of response, while lower FC in the dorsal DMN was predictive of nonresponse. Pei et al. (2020) used an SVM classifier based on the FC of 14 priori brain regions and predicted antidepressant response with a BAC of 81%. Notably, a model trained using whole-brain features achieved the same accuracy (81%).

Similar to Korgaonkar et al. (2020), Braund et al. (2022) used iSPOT-D data (SSRI/SNRI treatment) to investigate intrinsic FC networks that characterized neuroticism in 229 MMD patients, and using an SVM predicted treatment response with a BAC of 74%. Greater connectivity within and between the salience, executive control, and somatomotor brain networks was associated with higher baseline neuroticism. Irrespective of treatment type, increased network activity was predictive of poorer treatment outcomes that was not mediated by baseline neuroticism. Goldstein-Piekarski et al. (2018) also used iSPOT-D data, to predict treatment remission using connectivity within the DMN, with a focus on the posterior cingulate cortex (PCC). Connectivity between the PCC and ACC/mPFC together was predictive of remission (82% accuracy), whereby nonremitters showed relative hypoconnectivity compared with remitters, who showed intact connectivity similar to that of controls. Differential prediction of remission using PCC connectivity did not survive correction.

Cash et al. (2019) significantly predicted rTMS response using rsFC in the DMN and affective network (AN), as well as BOLD signal power and Week 1 clinical response (*r* = 0.68, *p* < 0.001, 93% accuracy). However, some significant relationships between individual features and treatment outcome were only observed once participants with the lowest treatment outcome (<0% change) were omitted. Hopman et al. (2021) attempted to replicate previous findings that stronger dlPFC-sgACC anticorrelated connectivity was associated with rTMS response; however, the authors could not confirm this relationship. Instead, using seed-based features of the left dlPFC (rTMS target) and sgACC, the authors predicted rTMS response with 89% accuracy, finding that greater connectivity disruptions involving the central executive network was associated with poorer response.

## GENERATIVE MODELING AND GENERATIVE EMBEDDING

Compared with the studies reviewed so far, generative embedding approaches have the potential to better capture disease mechanisms and provide more interpretable treatment response predictions (Brodersen et al., 2011; Frässle et al., 2018). Generative embedding incorporates generative models of information processing dynamics in the brain. By fitting these models to (brain or behavioral) data, one can effectively reduce the dimensionality of the raw data to a handful of highly informative model parameter estimates—that is, mechanistically interpretable features. These parameter estimates are then used as input features in machine learning algorithms to predict treatment response. Depending on how well these models capture mechanisms relevant for treatment response prediction, this approach could improve not only the interpretability of the predictions but also their accuracy. However, generative embedding is yet to be fully utilized in the context of treatment response prediction in MDD. In this section, we would like to highlight several lines of research employing generative modeling approaches that have been used to study MDD but have not been applied to treatment response prediction.

### Generative Models of Brain Data

One way to model the information processing dynamics in the brain is by using dynamic causal modeling (DCM), which allows for estimation of directed interdependencies (i.e., effective connectivity) among multiple brain regions (Friston et al., 2019; Stephan & Friston, 2010). Unlike functional connectivity, which describes temporal correlations in BOLD responses across brain regions, effective connectivity rests on a generative model, which specifies directed relationships between populations of neurons (Friston, 2011). DCM is thus able to account for asymmetries in forward and backward connections (Bastos et al., 2012; Felleman & Van Essen, 1991; Markov et al., 2014), which creates a possibility for a more detailed characterization of disease mechanisms. Furthermore, recent studies using DCM for electrophysiological data were able to incorporate microscale details such as the conductance of specific receptor populations (Gilbert et al., 2016; Moran, Symmonds, Stephan, Friston, & Dolan, 2011; Schöbi et al., 2021; Symmonds et al., 2018), demonstrating the potential of DCM for multilevel description (from micro- to macroscale) of disease mechanisms. Estimating receptor densities in vivo from noninvasive EEG recordings would be a major step towards linking psychiatric symptoms to the mechanisms of action of pharmacological interventions that target specific neurotransmitter systems.

While many studies have applied DCM for studying mechanisms underlying MDD (Li et al., 2018), so far very few have used it for treatment response prediction. Vai et al. (2016) used effective connectivity measures obtained from DCM to investigate treatment response to escitalopram. Pretreatment effective connectivity during emotional face processing was found to discriminate nonremitters from remitters and controls after six weeks of treatment. Nonremitters showed reduced endogenous connectivity from the amygdala to the ventrolateral PFC and to the ACC, and increased modulation of the ACC to the amygdala when processing fearful faces. However, these results were obtained by performing a series of *t* tests and there was no analysis of how accurately these effects could predict treatment at the individual level.

Frässle et al. (2020) is the only study to apply a generative embedding approach for predicting illness course in MDD. Unlike the studies reviewed so far, this study aimed to predict long-term (up to two years) trajectories of MDD in a naturalistic cohort (participants were receiving mixed treatments), and thus it is not directly comparable, but it serves to illustrate how generative embedding could be applied for treatment response prediction. In this study, illness course was defined by dividing participants into three different groups: remitted (rapid remission); improved (slow remission); and chronic (treatment-resistant). Pretreatment fMRI data recorded during an emotional face perception task was used for predicting illness course. Six regions of interest (ROIs) were selected based on their association in the literature with the extended face perception network: bilateral occipital face area, fusiform face area, and amygdala. The best model allowed emotion processing to modulate forward and backward intra- and interhemispheric connections among homotopic brain regions. Using effective connectivity parameters as features, SVM predicted chronic versus remitted groups with BAC of 79%, and improved versus remitted groups with BAC of 61%. Importantly, this performance exceeded that of conventional non-generative methods that used functional connectivity or local activation (computed from the same network of ROIs) as features for classification; these did not result in above-chance performance.

### Generative Models of Behavioral Data

While generative models of brain dynamics can help us explain neural data, they fall short of explicitly linking these dynamics to behavior. Given that relevant clinical symptoms manifest in behavior, explaining observable behavior is an important consideration for generative models. In the research setting, MDD has been associated with deficits in value-based decision-making, especially in tasks involving reinforcement learning and expectations about the future (Eshel & Roiser, 2010; Mukherjee, Lee, Kazinka, Satterthwaite, & Kable, 2020; Must, Horvath, Nemeth, & Janka, 2013). Not surprisingly, one of the most popular frameworks for modeling these aspects of behavior in MDD has been reinforcement learning (RL), which models adaptive decision-making in the face of rewards and punishments (Chen, Takahashi, Nakagawa, Inoue, & Kusumi, 2015; Huys, Pizzagalli, Bogdan, & Dayan, 2013). The central variable in RL is the reward prediction error (RPE)—the difference between expected and observed reward/punishment—which guides learning of value of different stimuli, which in turn guides actions. Importantly, RL modeling approaches make it possible to study reward processing in a lot more detail and investigate how different elements of the decision-making process such as RPE, expected value (“wanting”), reward sensitivity (“liking”), learning rate, memory of previous reinforcement, noisiness of action selection, and so on, might be implicated in MDD and how they might relate to different MDD subtypes (Chen et al., 2015; Robinson & Chase, 2017; Rupprechter, Stankevicius, Huys, Steele, & Seriès, 2018). Crucially, even though RL models are fitted to behavioral data, the computational processes described by them can then be used to investigate brain activity specific to this process by including model variables as regressors in a general linear model (GLM), which in neuroimaging data analysis, has been known as **model-based fMRI** (Gläscher, Daw, Dayan, & O’Doherty, 2010; Katahira & Toyama, 2021; O’Doherty, Hampton, & Kim, 2007).

To date, only one study has applied RL model-based fMRI for treatment response prediction in MDD. Queirazza, Fouragnan, Steele, Cavanagh, and Philiastides (2019) aimed to obtain mechanistically meaningful fMRI predictors of response to computerized cognitive behavioral theraphy (cCBT) based on pretreatment brain activity during a probabilistic win/loss reversal-learning task. The data were analyzed by first fitting an RL model to the behavioral data to estimate trial-wise RPEs. Next, RPEs, weighted by a dynamic learning rate, were used as regressors in a GLM when analyzing the fMRI data. Finally, the resulting regression coefficients were used to predict cCBT treatment response using an SVM, RVM, and logistic regression. All classifiers showed comparable performance, with the SVM yielding the best performance (BAC 72%). Neural activity encoding-weighted RPEs in the right striatum and right amygdala were the most discriminative features of treatment response, with greater pretreatment activity predicting better response. The authors suggested that greater neural signaling of the weighted RPE might make cognitive restructuring practiced during cCBT more effective, fostering more balanced beliefs about the self and the world.

MDD can also be understood within a more general computational framework of Bayesian inference (Badcock, Davey, Whittle, Allen, & Friston, 2017; Barrett, Quigley, & Hamilton, 2016; Chekroud, 2015; Huys, Daw, & Dayan, 2015; Kube, Schwarting, Rozenkrantz, Glombiewski, & Rief, 2020; Paulus & Angela, 2012; Stephan et al., 2016). Central to Bayesian accounts of decision-making is the observation that external (world) states and internal (bodily) states cannot be directly observed and must be inferred from ambiguous sensory information. Similarly, different action policies and their consequences also carry varying degrees of uncertainty. All these types of uncertainty shape one’s expectations of rewarding or aversive outcomes (cf. reinforcement learning), but even more importantly, they determine how information is sampled (via action selection and attention) and to what extent the resulting positive or negative experiences are integrated into one’s model of the world. Within this framework, deficits in value-based decision-making in MDD can be cast as a biased construction of internal and external states, which results in a maladaptive positive feedback loop involving one’s model of the world, action selection, and mood. The biases themselves could result from a miscalibration of precision (the inverse of uncertainty) associated with prediction errors (PEs)—which, similarly to RPEs, represent the difference between expected and experienced sensory input and guide learning (Badcock et al., 2017; Barrett et al., 2016; Kube et al., 2020). For instance, attenuation of PEs would make one immune to changing one’s negative beliefs in the face of disconfirming evidence (Barrett et al., 2016; Kube et al., 2020). On the other hand, increased precision of PEs for social contexts may increase sensitivity and attention to interpersonal cues and could lead to social withdrawal and anhedonia (Badcock et al., 2017). Finally, in the context of interoception, a miscalibration of the precision associated with PEs would lead to a disruption of allostasis (i.e., the brain’s ability to anticipate and flexibly adapt to changing metabolic needs), which could explain fatigue and inflammation observed in MDD (Barrett et al., 2016; Stephan et al., 2016). Relevantly, recent preliminary findings by Hough et al. (2021) indicate that pretreatment allostatic load and metabolic dysregulation might be predictive of SSRI response. Thus, adopting a Bayesian framework allows for integration of brain and behavior as well as the body and physiological states. This potentially affords a much more comprehensive picture of MDD. So far, to the best of our knowledge, no studies have applied these models for treatment response prediction.

## OTHER REMAINING CHALLENGES

While theory-driven generative models have the potential to improve our understanding of MDD as well as capture interpretable and discriminative features for treatment response prediction, multiple other challenges remain.

### Treatment Response Prediction Versus Differential Treatment Response Prediction

Most of the studies reviewed in this paper have focused on building a model that can predict treatment response for a single intervention. In clinical practice, a more useful prediction would be *differential* treatment response prediction, indicating which of *several* available treatments is the most likely to lead to improvement for a given patient (Dunlop & Mayberg, 2014; Perlman et al., 2019). This of course could be determined by applying individual models for each treatment and then aggregating the results to derive differential prediction. However, if different models have to rely on different data modalities and require separate validations, this would be much more time- and resource-intensive. Therefore, a single model that can reliably predict treatment response to multiple treatment options would be much more useful in practice.

Several of the reviewed studies did involve multiple treatments, but differential treatment response prediction remains very limited. A tbfMRI study by Tozzi et al. (2020) found that greater functional connectivity between both dlPFC-SMG and SMG-MTG during behavioral inhibition was associated with response to SSRI sertraline, whereas lower connectivity was associated with response to SNRI venlafaxine; however, their model failed to predict response to SSRI escitalopram. Another tbfMRI study by Williams et al. (2015) found amygdala hyporeactivity to subliminal happy and threat expressions to predict treatment response to escitalopram, sertraline, and venlafaxine, while amygdala hyperreactivity to sad expressions specifically predicted nonresponse to venlafaxine. Investigating the same three treatments, two rsfMRI (Goldstein-Piekarski et al., 2018; Korgaonkar et al., 2020) and one rsEEG (Rajpurkar et al., 2020) studies, were only able to predict treatment response across the treatments but not differential treatment response. Some studies involving multiple treatments did not investigate differential treatment response despite having large enough sample sizes (Braund et al., 2022; Cook et al., 2020; Pei et al., 2020), while others were limited by too small samples (Crane et al., 2017; Jaworska et al., 2019; Ju et al., 2020; Khodayari-Rostamabad, Reilly, Hasey, de Bruin, & MacCrimmon, 2013; Patel et al., 2015; Rabinoff, Kitchen, Cook, & Leuchter, 2011; Sikora et al., 2016).

For more clinically relevant results, future studies should focus on establishing predictions of differential treatment response and include a wider range of treatments (e-psychotherapy, ECT, rTMS, first-line antidepressants, rapid-acting antidepressants, psychedelic therapy). Bearing in mind the heterogeneity of MDD, this would be of more benefit than comparing very few treatments with similar mechanisms of action (e.g., escitalopram vs. sertraline). This, however, would require large samples and multisite studies, which can be practically challenging. One of the largest current projects, iSPOT-D (Williams et al., 2011), aims to recruit *N* = 672 per treatment arm but includes only three treatments (escitalopram, sertraline, and venlafaxine).

### Defining Outcomes

#### Problems with dichotomization.

The majority of studies reviewed here focused on predicting either treatment response or remission by dichotomizing the reduction in symptoms of depression—with *response* often defined as ≥50% reduction in symptoms from the baseline and *remission* defined as reaching a score below some low threshold, such as ≤7 for HAMD-17. This is rather problematic because dichotomizing continuous variables leads to a loss of information and thus a loss of statistical power (Altman & Royston, 2006; Maxwell & Delaney, 1993). For example, a patient with 49% reduction in symptoms will be considered to be the same as a patient with 0% reduction (both being “nonresponders”) but categorically different from a patient with 50% reduction in symptoms (a “responder”). Ignoring this within-group variability could lead to false inferences about the features that are predictive of treatment response (Austin & Brunner, 2004). Even more importantly, such coarse categorization undermines the very possibility of accurately predicting treatment outcomes.

Thirteen studies have circumvented this problem by applying regression models for predicting the symptom improvement on a continuous scale (see Tables 1 and 2), and one study (Rajpurkar et al., 2020) predicted improvement in individual symptoms, rather than summed symptom scores. Although the majority of studies produced strong results (median *R*^2^ = 0.24), it is not straightforward to compare them with the classification accuracy reported in the other studies that dichotomized symptom scores. In principle, predicted symptom scores could be converted to classification accuracy by post hoc dichotomization. This was exemplified by two studies, whereby the model’s regression outputs were dichotomized post hoc to obtain remission (Nguyen et al., 2019; Sun et al., 2020) prediction accuracy. Converting symptom improvement predictions into classification accuracy might also have benefits in clinical applications.

Another consideration when modeling symptom improvement is whether baseline depression severity should be included as a predictor or used to derive the predicted outcome (the difference between baseline and posttreatment depression severity). In some cases, these options can lead to diverging results (Lord, 1967; Tu, Gunnell, & Gilthorpe, 2008). However, when the baseline exhibits collinearity with other predictors of interest—as we might expect to be the case for depression severity and brain activity—including baseline as a predictor has been shown to lead to biased results (Castro-Schilo & Grimm, 2018; Farmus, Arpin-Cribbie, & Cribbie, 2019; Liu, Lu, Mogg, Mallick, & Mehrotra, 2009). In line with this, all symptom improvement studies reviewed here did not include baseline severity as a predictor. Note that when predicting dichotomized outcomes, baseline severity is also implicitly included in the outcome definition for the classification of responders versus nonresponders, because treatment response is usually defined as percentage change scores (e.g., 50% symptom improvement compared with baseline). Conversely, in the case of remitters versus nonremitters, the baseline does not feature at all as remission is defined as absence of clinically relevant symptoms at follow-up regardless of baseline symptom severity. However, among the reviewed studies that predicted dichotomized outcomes, the vast majority tested and reported no differences in baseline scores between the groups, which makes the considerations of baseline effects not relevant for the interpretation of the results. When baseline differences do exist in the context of predicting remission, including baseline among predictors should be considered (e.g., Karim et al., 2018).

#### Predicting improvement of individual symptoms to address MDD heterogeneity.

It is also worth considering whether using the overall improvement across symptoms is sufficiently informative for predicting treatment outcomes. MDD is a broad category, and diagnosed individuals display diverse symptom profiles, with some sharing no symptoms in common (E. Fried, 2017; E. I. Fried, Nesse, Zivin, Guille, & Sen, 2014; Goldberg, 2011). This heterogeneity is also reflected in the most commonly used MDD rating scales, such as HAMD (Hamilton, 1967), MADRS (Montgomery & Åsberg, 1979), BDI (Schwab, Bialow, Clemmons, Martin, & Holzer, 1967), and QIDS (Rush et al., 2003); these instruments differ considerably in their item content because they place different emphasis on different MDD symptoms (E. I. Fried, 2017). Developing models that can predict treatment effects on each individual symptom, thus, would make it possible to account for heterogeneity in MDD and would prevent problems of comparing study findings based on total depression scores that are derived from instruments that overlap but that are not interchangeable. One exemplar study reviewed here (Rajpurkar et al., 2020) addressed this issue by building a model to predict the change in each of the 21 items in HAMD-21 based on rsEEG data. The authors used gradient-boosted decision trees (GBDT), which allows for modeling of nonlinear associations. Their model achieved a concordance index of ≥0.8 on 12 out of the 21 symptoms (*R*^2^ of 0.32–0.7). These results are encouraging and suggest that prediction of treatment effects on individual MDD symptoms is feasible.

#### Patient-centered definition of outcomes: Functional recovery and quality of life.

Another consideration is that the widely adopted depression scales used to assess symptom severity do not capture all relevant aspects of treatment outcomes. From a patient’s point of view, symptom resolution is only one factor in determining remission from depression. Positive aspects of mental health (Zimmerman et al., 2006), functional recovery (Greer, Kurian, & Trivedi, 2010; Lam, Parikh, Michalak, Dewa, & Kennedy, 2015; Oluboka et al., 2018), and quality of life (QoL; IsHak et al., 2011) are just as important. MDD-related functional impairment can span multiple domains such as occupational, social, physical, and cognitive functioning, for which both objective and subjective measures exist. QoL highly overlaps with functional impairment measures but often assesses well-being across a wider variety of domains, including emotional well-being and overall life satisfaction, and is often based on subjective self-rating of these domains (Endicott, Nee, Harrison, & Blumenthal, 1993; Frisch et al., 2005; Leon et al., 1999). The impact of MDD on the aforementioned domains is not always well captured by the scales designed to assess depressive symptoms, while remission of depressive symptoms alone does not necessarily lead to full functional recovery (Habert et al., 2016) and does not fully restore QoL (IsHak et al., 2013; Morton et al., 2021). Relatedly, the narrow assessment of MDD symptoms does not take into account treatment side effects, which are very common for antidepressant drugs (Read & Williams, 2018). Only one of the reviewed studies (Goldstein-Piekarski et al., 2016) focused on the prediction of functional remission.

Understanding the differential effects of available treatments on these measures is therefore an important research direction. A meta-analysis comparing CBT and SSRI treatments found similar effects on QoL; however, depression improvement was associated with increased QoL only in the CBT group (Hofmann, Curtiss, Carpenter, & Kind, 2017). Another recent study by Fischer et al. (2021) found depression symptom and QoL improvements to be associated with partially distinct changes in functional connectivity of reward neurocircuitry, which was also differentially affected by different antidepressants (sertraline, venlafaxine-XR, and escitalopram). Finally, Koshiyama et al. (2020) found rsEEG beta band power at baseline to be strongly correlated with QoL outcomes at the three-year follow-up, and this correlation was independent of a reduction in symptoms. Following this line of research, to achieve a more complete assessment of treatment outcomes, future studies should consider including functional recovery and QoL among the predicted treatment outcomes.

### Reliability of Prediction Accuracy: Sample Size and Validation

Based on the American Psychiatric Association recommendations, treatment response prediction accuracy of at least 80% would be considered to have clinical utility (Botteron et al., 2012). Most of the reviewed studies report prediction accuracy above 80%, and thus would appear to have sufficient accuracy. However, these numbers are likely to be positively biased. The reviewed studies varied considerably in sample size as well as validation methods (see Tables 1 and 2)—both of which affect the generalizability of the reported prediction accuracy (Gillan & Whelan, 2017; Janssen, Mourão-Miranda, & Schnack, 2018; Kelly et al., 2019; Yarkoni & Westfall, 2017).

A substantial number of studies were not included in this review because of a lack of validation methods. Such studies reported classification accuracy for the data that the classifier was trained on; this leads to overfitting and poor performance on unseen data (Gillan & Whelan, 2017). To improve generalizability, the majority of studies used internal validation, namely LOOCV or k-fold CV, which trains the classifier on all but a subset of patients and uses the left-out patients for testing model accuracy—repeating this process by permuting all data. However, for the median sample size of the reviewed studies, *N* = 47, using LOOCV can still lead to accuracy errors of up to ∼15% (Varoquaux, 2018), with k-fold suffering from similar problems (Vabalas, Gowen, Poliakoff, & Casson, 2019). The least biased method, nested CV, adds an additional layer of validation for testing the model on an unseen portion of the data (Vabalas et al., 2019; Varma & Simon, 2006; Varoquaux et al., 2017). Nested CV is especially important for data-driven feature selection (i.e., filtering) where certain features are removed if they are not associated with the target variable (e.g., treatment response). If done for the whole dataset, this is problematic because it leads to positively biased prediction results. To prevent data leakage, all data preprocessing (e.g. feature selection, imputation, hyperparameter tuning) should be embedded within the cross-validation procedure. Nested CV was used by only six of the reviewed studies (Frässle et al., 2020; Hopman et al., 2021; Khodayari-Rostamabad, Reilly, Hasey, de Bruin, & MacCrimmon, 2010; Leaver et al., 2018; Nguyen et al., 2019; Patel et al., 2015). However, for sample sizes between 100 and 200, which a few reviewed studies had (see Tables 1 and 2), the error associated with the reported accuracy could still be up to 8–10%, regardless of the CV method used (Varoquaux, 2018).

Irrespective of the internal validation used, test cases are randomly selected from the same dataset on which the training is performed. The generalizability of such results can thus be undermined by sample- or site-specific confounds. A good way to protect from site-specific confounds is to perform leave-one-site-out CV (LOSOCV), which was done by two multisite studies: Zhdanov et al. (2020), performing LOSOCV across four different sites (*N* = 122), and Tian et al. (2020), performing LOSOCV across three different sites (*N* = 106). To properly test a model’s performance, an independent external dataset, which may differ with respect to clinical assessments (e.g., HAMD vs. MADRS), inclusion criteria (e.g., as it relates to comorbidities), and data acquisition/preprocessing parameters, is required. For example, the model by N. Bailey et al. (2019), using rsEEG to predict rTMS response in treatment-resistant depression (*N* = 50) and achieving BAC 86% with k-fold CV, was not predictive of treatment response when tested on an independent dataset of *N* = 193 (N. W. Bailey et al., 2021). Similarly, Drysdale et al. (2017) found four depressive subtypes from rsfMRI data, one of which was associated with large response rates to rTMS treatment; however, these subtypes could not be reproduced in an independent study (Dinga et al., 2019).

The ultimate test of treatment response prediction will require prospective validation studies to assess the actual improvement of outcomes following prediction-based treatment selection as compared with treatment-as-usual (van der Vinne et al., 2021). Such studies will also require careful consideration of how treatment response predictions can be most optimally integrated into the clinical workflow (Kelly et al., 2019).

Two conclusions can be drawn from the generalizability issues discussed above. First, for more informative results, studies should employ validation methods that are less biased, such as nested CV. While more practically challenging, to further increase generalizability of results, studies need to perform external validation of their model predictions. Second, small sample sizes create a bottleneck for any model validation efforts. Thus, multisite studies with larger sample sizes (>100) will be crucial for producing sufficiently reliable results. Currently, such initiatives include iSPOT-D (Williams et al., 2011), which aims to recruit *N* = 672 per treatment arm; Canadian biomarker integration network in depression (CAN-BIND; Kennedy et al., 2012), aiming to recruit *N* = 290; and EMBARC study (Trivedi et al., 2016) with *N* = 160. Larger samples would also help address another issue limiting generalizability: sample bias. The reviewed studies varied in their inclusion criteria, both related to comorbidities (anxiety, bipolar, substance use disorders, etc.) and MDD progression (drug-naive vs. treatment-resistant vs. late-life depression). Larger samples with more inclusive criteria would be more representative of the broader MDD population, where comorbidities are common (Steffen, Nübel, Jacobi, Bätzing, & Holstiege, 2020).

## CONCLUSION

Because of the heterogeneity in MDD presentation, etiology, and trajectory, the selection of an appropriate treatment by clinicians is a challenging task. This review has detailed the emerging use of computational models for individual treatment response prediction in MDD, highlighting methodological differences along the data-driven and theory-driven spectrum. Although both approaches have shown promising results, multiple challenges remain. Here we argued that a promising research direction for improving interpretability and, potentially, the accuracy of model predictions is theory-driven generative models, which allow for inference on disease mechanisms. Furthermore, we identified several other methodological limitations related to treatment outcome definition and validation of model predictions. The success of translating these tools to clinical practice will depend on carefully designed external validation studies with diverse patient samples and patient-centered outcome measures (see Box 1).

**Box 1.** Future directions Obtaining clinically relevant features:

- Theory-driven generative models of brain activity and behavior can help extract more interpretable and more discriminative features for treatment response prediction.
- A comprehensive model of the mechanisms underlying MDD symptomatology could be built using the hierarchical Bayesian inference framework.

Differential treatment response over treatment response prediction:

- Including more than one and ideally several different treatments would allow for the prediction of *differential* treatment response, which is clinically more useful than predicting response to a single treatment only.

Defining treatment outcomes:

- Predicting symptom improvement on a continuous scale, rather than dichotomized remission or response outcomes, can increase statistical power and prediction accuracy.
- Predicting improvement in individual symptoms can help avoid confounds associated with the diverse symptom profiles of the individuals diagnosed with MDD.
- Going beyond symptom reduction and including functional recovery and quality of life as relevant outcomes can help achieve more patient-centered and thus more relevant predictions of treatment outcomes.

Validating computational models:

- To produce more reliable and generalizable results, more robust validation methods need to be employed. At the very least, nested cross-validation should be used for internal model validation. To further improve generalization, external validation across more than one site is needed.
- Larger sample sizes (*N* > 100) are essential for enabling such validation techniques and ensuring the representativeness of the MDD population.

## AUTHOR CONTRIBUTIONS

Povilas Karvelis: Conceptualization; Formal analysis; Investigation; Methodology; Visualization; Writing – original draft; Writing – review & editing. Colleen E. Charlton: Conceptualization; Formal analysis; Investigation; Methodology; Visualization; Writing – original draft; Writing – review & editing. Shona G. Allohverdi: Conceptualization; Investigation; Writing – original draft; Writing – review & editing. Peter Bedford: Conceptualization; Investigation; Writing – review & editing. Daniel J. Hauke: Conceptualization; Investigation; Writing – review & editing. Andreea Diaconescu: Conceptualization; Funding acquisition; Methodology; Project administration; Supervision; Writing – review & editing.

## FUNDING INFORMATION

Andreea Diaconescu, Krembil Foundation (https://dx.doi.org/10.13039/501100004089), Award ID: 1000824. Daniel J. Hauke, Schweizerischer Nationalfonds zur Förderung der Wissenschaftlichen Forschung (https://dx.doi.org/10.13039/501100001711), Award ID: 200054.

## TECHNICAL TERMS

Supervised learning: A set of techniques for inferring a function that maps input features (e.g., neuroimaging features) to output labels (e.g., treatment response) based on training examples of feature-label pairs.
Unsupervised learning: A set of techniques for finding patterns in the distribution of features (e.g., neuroimaging measures) without using labels (e.g., treatment response).
Interpretability: The degree to which a user can comprehend why certain predictions have been made by a predictive model.
Task-based neuroimaging: Measures brain activity that is functionally involved in a specific cognitive or behavioral task.
Resting-state neuroimaging: Measures brain activity and regional interactions that occur in the absence of a stimulus or a task (“at rest”).
Generative embedding: The use of features extracted via a generative model as input for supervised or unsupervised machine learning algorithms.
Cross-validation: A validation technique used to estimate model performance by training the model on a subsample of data and validating model predictions on the remaining sample. This process is often repeated by permuting the data.
External validation: A technique that validates a model trained on one dataset on an independently acquired dataset to determine the model’s generalizability and reproducibility.
Model-based fMRI: Extracts features of latent cognitive mechanisms using theory-driven generative models of behavioral data and uses those features as regressors in GLM-based fMRI analysis to find neural correlates.
